# The Long-Term Effect of Famine Exposure on Cognitive Performance: Evidence from the 1959–1961 Chinese Famine

**DOI:** 10.3390/ijerph192416882

**Published:** 2022-12-15

**Authors:** Han Zhang, Wing Chung Ho

**Affiliations:** 1 Department of Social Work and Social Administration, University of Hong Kong, Hong Kong SAR, China; 2 Department of Social and Behavioural Sciences, City University of Hong Kong, Hong Kong SAR, China

**Keywords:** Chinese Famine, long-term impact, cognitive performance, difference-in-differences

## Abstract

We examined the long-term impact of the 1959–1961 Chinese Famine on the survivors’ cognitive performance in this study. Using data from the 2010 China Family Panel Study, our cohort comparison analysis showed that people who experienced the famine in early childhood (aged 1–3) had a lower score on a vocabulary test and that those who were exposed to the famine in utero did not differ from those born after the famine, probably due to positive selection for the in utero survivors. To deal with the problem of the lack of a comparable control group, we further applied a migrant–stayer comparison approach, with data from the 2016 China Family Panel Study and the 2017 Hong Kong Panel Study of Social Dynamics, to examine the effects of famine exposure at different life stages on adult cognition. We compared the people who stayed in Guangdong with the people who crossed the border to Hong Kong before the famine. The results showed that Guangdong stayers who experienced the famine when they were aged 1–18 had worse performance in immediate word recall. The findings suggested that exposure to malnutrition during childhood has long-term adverse effects on cognitive performance.

## 1. Introduction

Prenatal or postnatal exposure to malnutrition has lasting effects on individuals’ health conditions. Previous studies often used famines, such as the Dutch Famine (1944–1945), the siege of Leningrad (1941–1944), and the Great Leap Forward Famine (1959–1961) in China, as empirical settings to examine the impacts of early-life nutritional deprivation on adult physical and mental health [1,2,3,4,5,6,7,8].

Cognitive performance is one of the significant outcomes that could be affected by nutritional deprivation. Cognitive performance is shaped by genetic and environmental exposures across the lifespan, with the period from conception to the age of 3 being a golden opportunity for brain development [9,10,11]. Cognitive performance also plays an important role in determining individuals’ socioeconomic status, health, and subjective well-being. The decline of cognitive functioning in the elderly is detrimental to their quality of life and causes higher demands on medical care, as well [12,13]. As population aging has become an increasingly urgent challenge across the globe, research on the cognitive performance of middle-aged and elderly people bears important academic and policy implications.

While the impacts of exposure to famine on cognitive performance have been extensively examined, the vast majority of famine-based studies were in the scope of the foetal origins literature [14], focusing on prenatal and early-childhood exposure to malnutrition. The empirical findings are mixed. The 1983 famine in Ghana, for example, was documented as having had long-term cognitive effects on survivors, and the famine impacts on intelligence test scores were most severe for those who were aged two or below during the famine [15]. An early gestation exposure to the Dutch Famine was associated with poorer selective attention performance at ages 56 to 59 [16]. Other studies on the Dutch Famine, however, found no significant differences in adult cognitive outcomes, such as cognitive functioning test scores and intellectual disability, between people who were exposed to famine in utero and those who were not [17,18]. The insignificant associations between famine exposure and survivors’ later-life cognitive performance were thought to be a result of selective survival (that foetuses impaired by nutritional deprivations would not survive, and survivors were positively selected) and/or compensatory experience (that postnatal experience such as investments in education might have compensated for the negative effects of early life malnutrition) [18].

The foetal origins literature addressed a very important aspect, yet only part, of the story of famine impacts. Famine experience during childhood and early adulthood matters, as well [13,19]. Apart from the critical period of brain development under age 3, cognitive abilities also develop in responses to environmental stimuli during childhood and early adulthood. For instance, episodic memory, a critical component of cognitive function, develops significantly in childhood and adolescence [20].

In this study, we aim to advance the literature by examining the effects of prenatal, childhood, and early adulthood exposure to the Chinese Famine on the survivors’ later lives cognitive abilities. The 1959–1961 Chinese Famine was a nationwide disaster characterized by varied famine severity in different regions in China. It caused approximately 30 million excess deaths, with a mortality rate of over 3.0% [21,22]. The impacts of the Chinese Famine have also been extensively studied, covering a wide range of topics such as body mass index, self-rated health, hypertension, and mental health [19].

With relevant data on cognition becoming available, more attention has been paid to empirical investigations on exposure to the Chinese Famine at different stages of early life and its associations with various dimensions of cognitive performance in adulthood [11,23,24,25]. Using clinical data from subjects recruited from local hospitals in multiple regions, one study found negative associations between famine exposure at different early life stages and specific cognitive performances. The findings show that exposure during the foetal period (relative to the unexposed group) related to lower scores on the Mini-Mental State Examination; exposure during late childhood (7–9 years old during the famine) was linked to lower scores on the Montreal Cognitive Assessment; and exposure during mid- (4–6 years old during the famine) to late-childhood related to poorer performance on the Stroop colour and word test [11]. Another study, using a clinical sample recruited from a public hospital in Shanxi province, confirmed the negative associations between famine exposure in the foetal and multiple childhood periods and cognitive decline in adulthood and further argued that the nutrition consumption pattern of adulthood played an important role in the link [24].

While the findings of the studies mentioned above were subject to generalizability limitations, Xu et al., (2018) used data from a nationally representative survey, the China Health and Retirement Longitudinal Study, to examine the effects of famine exposure during prenatal and early postnatal life on the midlife cognition of rural residents [25]. They conducted a difference-in-differences (DID) analysis using birth cohorts and regional variations in famine severity and found that the 1961 cohort had worse performance than the unexposed 1963 cohort in the Telephone Interview for Cognitive Status and general cognition in their early 50s. Their empirical analysis further suggested that there was a mixture of cognition-damaging effects and positive mortality selection effects, resulting in observations of positive, negative, and no effects of famine exposure for different birth cohorts of survivors.

The DID strategy with representative data serves as a better approach to identifying the causal effect of famine exposure on cognition and has been frequently used in studies on other long-term consequences of nutritional deprivation, as well [1,26]. However, the lack of a genuine control group was the one major drawback of the previous literature on the Chinese Famine [19], as the famine was a nationwide disaster of the top-down Great Leap Forward campaign, affecting people across mainland China [27,28].

In the current study, we examined the effects of prenatal, childhood, and early adulthood exposure to the Chinese Famine on survivors’ later life cognition. To fill the research gaps mentioned above, we first employed the commonly adopted cohort comparison approach to examine whether famine exposure in early childhood and the utero period had a lasting impact on survivors’ cognitive performance, using data from the 2010 China Family Panel Studies; then, we applied the migrant–stayer comparison approach introduced by Zhang, Song and Wu (2017), an innovative design with a control group, to examine how exposure to famine in childhood and early adulthood influenced adult cognition [19]. For the latter, we combined data from the 2016 China Family Panel Studies and 2017 Hong Kong Panel Studies to compare emigrants who moved to Hong Kong from Guangdong before 1959 (the control group) with residents who stayed in Guangdong and experienced the Chinese Famine (the exposed group).

Our findings showed no significant association between prenatal exposure to famine and cognitive performance. The survivors who experienced the famine in early childhood, however, were negatively influenced by the malnutritional experience. We also found that the people who stayed in Guangdong and experienced the famine when they were aged 1–18 had worse cognitive performance than the emigrants who moved from Guangdong to Hong Kong before the famine.

## 2. Materials and Methods

### 2.1. Research Design and Data

There are two analyses in our research designs. As mentioned, the first cohort comparisons design in mainland China involved data from the 2010 China Family Panel Study (CFPS). The CFPS is a nationally representative and longitudinal survey. The baseline wave of the CFPS was collected in 2010, and the sample covered a total of 14,960 households and 33,600 adults from 25 provinces in mainland China. For this analytical sample, we defined three cohorts for comparison: People born between 1959 and 1962, as the famine cohort, and people born between 1955 and 1958, as the pre-famine cohort, were the two exposed groups. People born between 1963 and 1966, the post-famine cohort, were the control group. The definitions of the cohorts restricted our analytical sample to 5513 respondents (All three cohorts comprised respondents who had rural origins (agricultural/rural household registration status at the age of 3)). We followed the commonly used DID approach to examine the effects of exposure to famine during the infant and foetal periods on later-life cognitive performance, with the interaction between famine cohorts and famine severity at the provincial level. We expected the pre-famine cohort would be more likely to have lower cognitive performance compared with those who did not experience the famine, while for those born during the famine period, their cognitive performance may not have been influenced by acute malnutrition.

Our second research design, the migrant–stayer comparison approach, went one step further to include emigrants from Guangdong to Hong Kong, who were exempted from the Chinese Famine as the control group, and to define people who remained in Guangdong and experienced the famine as the exposed group. In other words, we used cross-border migration from Guangdong to Hong Kong, the then British-ruled territory, as a quasi-experimental setting. Hong Kong and Guangdong are geographically adjoining areas sharing similar Cantonese culture, dialects, and lifestyles but have had distinct paths under their respective political systems. The emigrants who moved to Hong Kong before the famine were comparable to people who remained in Guangdong (the stayers) until the former moved out of the mainland. When mainland China experienced the famine between 1959 and 1961, the emigrants from Guangdong to Hong Kong were unexposed. We used the data from two representative surveys with similar sampling and questionnaire designs: the 2016 China Family Panel Studies (CFPS) and the 2017 Hong Kong Panel Study of Social Dynamics (HKPSSD). The HKPSSD is a citywide representative dataset with detailed information on place of birth, province of origin, and year of arrival [29]. Both the 2017 HKPSSD and 2016 CFPS datasets include measures of cognitive performance (All subjects gave their informed consent for inclusion before they participated in the study. The study was conducted in accordance with the Declaration of Helsinki and the protocol was approved by the Ethics Committee of (IRB0000105214010 and HKUST-6001-SPPR-08)).

Supplementary to the first cohort comparison approach, which examined the effects of foetal and infant malnutrition, the second migrant–stayer approach focused on famine exposure during childhood and early adulthood. We classified three birth cohorts: the 1923–1940 cohort (aged 19–36 when the famine began), the 1941–1958 cohort (aged 1–18 when the famine began), and the 1963–1968 cohort. The 1923–1940 cohort included two subgroups of respondents born between 1923 and 1940: (1) stayers in Guangdong who experienced the famine (from the CFPS data), and (2) emigrants who moved from Guangdong to Hong Kong before 1959 and who did not experience the famine (from the HKPSSD data). The 1941–1958 cohort included two similar subgroups of respondents, except that they were born between 1941 and 1958. The difference between the Guangdong-stayers and the Guangdong-Hong Kong-emigrants for each group could include both the famine effect and the migration effect. To address the concern of potential migration selectivity, we included the 1963–1968 cohort as a reference group (Note that, in the 2017 HKPSSD, the questions about cognitive performance were restricted to respondents aged 50 and above, which limited our analytical samples to people born in 1968 or before). The 1963–1968 cohort also included stayers and emigrants, without restrictions on the years of migration for the emigrants. As the 1963–1968 cohort was a post-famine cohort, none of the three subgroups was exposed to the famine. We conducted a difference-in-differences analysis by comparing the difference between the stayers and emigrants in the 1923–1940 and 1941–1958 cohorts, respectively (i.e., difference (1)), to the difference between the stayers and emigrants in the 1963–1968 cohort (i.e., difference (2)), to obtain an estimate of the famine effect (Our DID analysis followed Zhang, Song, and Wu’s (2017) approach [19]). Our analytical sample for the migrant–stayer comparison analysis included 130, 460, and 225 respondents for the 1923–1940, 1941–1958, and 1963–1968 cohorts, respectively (To be consistent with the first cohort comparison approach, the respondents in the analytical sample of the migrant–stayer comparison analysis were those with rural origins. The Guangdong stayers were respondents who had rural *hukou* status at the age of 3 in the CFPS data. The Guangdong emigrants were people who came from rural areas to Hong Kong according to the HKPSSD data). We expected that, for the 1941–1958 cohort who experienced famine during childhood, their cognitive performance would be more likely to be negatively influenced by the famine, whereas for those who experienced the famine in adulthood (1923–1940 cohort), this malnutritional experience would not have had a long-term impact on their cognitive performance.

### 2.2. Measures

#### 2.2.1. Key Independent Variables

As mentioned above, the key independent variable for the first cohort comparisons approach included three cohorts. They were the pre-famine (1955–1958), famine (1959–1962), and post-famine (1963–1966) cohorts, with the post-famine cohort as the reference group in the multivariate analysis. Apart from the cohort, the famine severity was used for the DID analysis. We used the death rates at the provincial level from Lin and Yang (2000) and applied Tan, Tan and Zhang’s (2014) approach to define famine severity as the difference between the highest mortality rate from 1959 to 1961 and the average mortality rate from 1956 to 1958 in each province [27,30]. The average mortality rate between 1956 and 1958 was relatively stable at 10.8 to 12 percent [27], and thus could be used as a reference for the death rate without famine. Famine severity was a continuous variable.

For the second migrant–stayer comparisons approach, the key independent variable, migrant status, included Guangdong (GD)-stayers and Guangdong to Hong Kong (GD-HK)-emigrants. We also included the birth cohort variable (1923–1940, 1941–1958, and 1963–1968) for the DID analysis.

#### 2.2.2. Cognition

For the cohort comparisons analysis, we used vocabulary test scores as our dependent variable to measure people’s cognitive performance. The validity of this measurement has been demonstrated by Huang, Xie and Xu (2016) [31]. In the CFPS, thirty-four Chinese characters were selected from primary and secondary language textbooks. The questions in the vocabulary test were arranged in ascending order of difficulty, and the questions varied by the highest education level of the respondents. Specifically, respondents with a primary educational level began with the first character, junior high school graduates started with the ninth character, and senior high school or above graduates started with the twenty-first character. The respondents were asked to recognize characters one-by-one until they could not identify three consecutive characters. The test score corresponded to the rank order of the last character that each of the respondents could answer correctly. If respondents failed to recognize any characters at his/her level, their test scores would be the rank order of their entry point minus one [31].

For the migrant–stayer comparisons analysis, cognitive performance was measured by a word recall test. Many studies have used the word recall test to measure individuals’ cognitive performance [25,32]. The word recall test included two variables. One was immediate word recall, for which respondents were given ten words randomly from the selected list (e.g., bird, river) to remember and were required to recall these words immediately. The test score corresponded to the number of words the respondents could recall. The other variable was delayed word recall, for which the respondents were required to recall the words after a five-minute delay, and the test score was also based on the number of words the respondent could recall. The two test scores were both continuous variables.

#### 2.2.3. Control Variables

For the first cohort comparisons analysis, we included age, gender, marital status, educational level, place of residence, parental educational level, and parental party membership as the control variables. Age was a continuous variable without any normalization. Gender was binary-coded 1 for males and 0 for females. Marital status was a dummy, with currently married coded as 1 and otherwise as 0. We classified educational levels into three categories: primary school or below (reference group), junior high school, and senior high school and above. Place of residence was a binary variable, with living in urban areas at the time of the survey coded as 1 and otherwise as 0. Parental educational level referred to either the father’s or mother’s educational level, whichever was higher, with three categories: primary school and below (reference group), junior high school, and senior high school and above. We also added parental party membership in this study, as the party membership played an important role in resource allocation, especially before the open-up and reform period [3]. We assumed that parents with party membership during the famine period may have influenced the family food resource distribution, to some extent. Parental party membership referred to whether the father or mother (or both) was a member of the Chinese Community Party, with yes coded as 1 and otherwise as 0.

For the second migrant–stayer comparisons analyses, we included age, gender, marital status, educational level, and place of residence as controls. The measurements of age, gender, marital status, and educational level are the same as in the cohort comparisons analysis. Note that, for emigrants in the HKPSSD sample, the place of residence was coded as urban.

### 2.3. Methods

We employed difference-in-differences (DID) methods for both the cohort comparisons analysis and the migrant–stayer comparisons analysis.

For the cohort comparisons analysis, the primary estimating equation was given in the following form:

*y* = *α* + *β*_1_*famine_cohort* + *β*_2_*edr* + *β*_3_(famine_cohort* *edr*) + *β*_4_*controls* + *ε*

where *y* was the outcome variable vocabulary test; the *famine_cohort* included 1955–1958, 1959–1962, and 1963–1966; and the *edr* was famine severity. The main interest of this analysis was the coefficient *β*_3_ of the interaction between the *famine_cohort* and *edr*, the DID estimate of the famine effect on later-life cognitive performance.

For the second migrant–stayer comparisons approach, we first conducted separate analyses comparing Guangdong-stayers (the exposed group) to Guangdong-Hong Kong-emigrants (the control group) for the 1923–1940, 1941–1958, and 1963–1968 subsamples, respectively. We then combined the 1923–1940 and 1963–1968 subsamples for a DID analysis to estimate the effect of the famine exposure during early adulthood (aged 19–36 when the famine started) on adult cognitive performance. Similarly, we combined the 1941–1958 and 1963–1968 subsamples for a DID analysis to estimate the effect of famine exposure during childhood (aged 1–18 when the famine started) on adult cognitive performance. The model specification for the DID analysis was:

*y* = α + *β*_1_*migrant_status* + *β*_2_*birth_cohort* + *β*_3_(migrant_status*birth_cohort) + *β*_4_*controls* + *ε*

where *y* was the test score, for either immediate word recall or delayed word recall; *migrant_status* included GD-stayers and GD-HK-emigrants; and *famine_cohort* included the 1923–1940 (or 1941–1958) and 1963–1968 cohorts. The coefficient, *β*_3_, of the interaction between *migrant_status* and *birth_cohort* was the estimation of the effect of famine exposure during early adulthood (or childhood) on cognitive outcomes.

## 3. Results

Appendix A Table A1 presents the summary statistics for the pre-famine, famine, and post-famine cohorts in the cohort comparisons analysis. The sample sizes of the pre-famine cohort (1955–1958), famine cohort (1959–1962), and post-famine cohort (1963–1966) were 1772, 1403, and 2397, respectively. For the measure of cognitive performance and the vocabulary test, the pre-famine cohort had the lowest scores, and the famine cohort seemed to have similar scores as the post-famine cohort. Yet the pre-famine cohort was also the oldest among the three cohorts. We also did the ANOVA and chi-squared tests for these three groups, and the results show that the differences in the vocabulary test among the three cohorts were significant. We then turned to the DID analysis for a better estimate of the effect of famine exposure.

Table 1 shows the results of the DID analysis with the interaction between the cohort and provincial famine severity, a common approach used in the literature. The coefficient of the interaction between the 1959–1962 cohort and famine severity was insignificant, whereas that between the 1955–1958 cohort and famine severity was significant for the vocabulary test. There were negative effects of exposure to famine during the infancy period on later-life cognition among the survivors. The insignificant result of the interaction between in utero famine exposure and famine severity might be due to a stronger positive selection of survivors. This result corresponded with our expectation that only survivors who were exposed to famine at ages 1–3 would show a negative influence on their cognitive performance resulting from malnutrition.

Appendix A Table A2 presents the descriptive statistics for the variables used in the migrant–stayer comparisons analysis. The mean score of the immediate word recall test was 2.5, 3.5, and 4.2, respectively, for the 1923–1940, 1941–1958, and 1963–1968 cohorts. The corresponding mean of the delayed word recall test was 1.5, 2.3, and 2.8. The differences in the two tests might be partly due to the disparities in age and educational level across the subsamples. For example, over 90 percent of those in the 1923–1940 cohort had an educational level of primary school or below, and only 4.6 percent had a level of senior high school or above. Yet the corresponding percentages were 73.9 and 11.7 for the 1941–1958 cohort, and 56.4 and 10.7 for the 1963–1968 cohort. To control the confounding effects of age and education as well as other variables, in Appendix A Table A2, we conducted a multivariate analysis of the subsamples in Table 2 and Table 3.

Table 2 presents the OLS regression results of immediate word recall for the three subsamples. Among the people born between 1941 and 1958 (who were aged 1–18 at the beginning of the famine), the score for immediate word recall was, on average, 0.646 points lower for the exposed group (i.e., GD-stayers who were exposed to the famine) than that of the control group (i.e., GD-HK-emigrants who were unexposed to the famine). The gap between the exposed group and control group was also negative among people born between 1923 and 1940 (who were aged 19–36 at the beginning of the famine), with a marginal significance level (*p* < 0.1). By contrast, the difference in immediate word recall between the GD-stayers and the GD-HK-emigrants in the post-famine cohort was positive (though statistically insignificant). The results in Table 2 show that the difference between the stayers and emigrants for the pre-famine cohorts (1923–1940 and 1941–1958) was not the same as the difference between the stayers and migrants where there was no famine effect. These results suggest that the GLFF had an adverse effect on an individual’s immediate word recall, especially for the 1941–1958 cohort.

Table 3 presents the OLS regression results on delayed word recall for the three subsamples. Among people born between 1923 and 1940, the difference in delayed word recall between the GD-stayers and the GD-HK-emigrants was −0.461, and that for the 1941–1958 subsample was −0.564. The gap between the stayers and emigrants for the post-famine cohort was also negative, though the difference was smaller (−0.397). The results in Table 3 show similar patterns for the three subsamples regarding the difference in delayed word recall for the three cohorts.

As mentioned above, a better estimate of the famine effect was achieved by running a DID analysis using the post-famine cohort as a reference. We combined the 1963–1968 subsample with the 1923–1940 and 1941–1958 subsamples, respectively, to generate two DID samples. The main interest was the interaction between the migrant status and birth cohort (1923–1940 vs. 1963–1968 or 1941–1958 vs. 1963–1968). The DID analysis provided more accurate estimates of the famine effects by considering the potential confounding factors of migration selectivity.

Table 4 presents the DID results on the immediate word recall test and delayed word recall test. For the people who were exposed to the famine when they were aged 1–18, their scores of immediate word recall were, on average, 0.806 lower than if they had avoided the famine (*p <* 0.05); the DID result of delayed word recall was negative, yet statistically insignificant. For the people who were exposed to the famine when they were aged 19–36, the DID result was not significant for either the immediate or delayed word recall. The DID results in Table 4 suggest that, for the people who were exposed to the Chinese Famine during the age of 1−18, there was a long-term adverse famine effect on their immediate word recall. However, no evidence shows that there was a long-term famine effect on people who were exposed to the famine during early adulthood. All the results provided further evidence of the long-term adverse effects of the GLFF on immediate word recall: the people who experienced malnutrition in the famine at age 1–18 (born in 1941–1958) had lower immediate word recall.

As an additional analysis, we conducted DID analyses for the 1923–1940 and 1941–1958 cohorts on the vocabulary test, using the first cohort comparisons approach. The results are presented in Appendix A Table A3. The model specification was the same as in Table 1, except that we used an alternative key independent variable, which included the 1923–1940, 1941–1958, and 1963–1968 (the reference) cohorts. The DID results show that there was an adverse effect of the famine exposure on the vocabulary test for the people who were aged 1–18 at the beginning of the famine. Yet there was no evidence of famine impact on the people who were exposed to the famine during early adulthood.

## 4. Discussion

This study examined the long-term effect of malnutrition during China’s 1959–1961 famine, a serious disaster in human history, on cognitive performance. Firstly, we conducted a cohort comparison analysis with the 2010 China Family Panel Studies survey data to examine whether prenatal and early childhood exposure to famine affected cognition. Our difference-in-differences (DID) results showed that the survivors who were born during 1955–1958 had poorer performance on the vocabulary test, suggesting that malnutrition during early childhood was negatively associated with later-life cognition. In utero exposure to famine, however, was not significantly associated with the adult vocabulary test results. Secondly, we combined comparable data from the 2016 CFPS and 2017 HKPSSD to compare the people who experienced the famine in Guangdong (the exposed group) with the migrants who crossed the border to Hong Kong before the famine (the control group) regarding adult cognitive performance. We used the innovative migrant–stayer comparison approach to examine the effects of famine exposure at different life stages on individuals’ cognition. Our results showed that the Guangdong-stayers who experienced famine at ages 1–18 (born in 1941–1958) had poorer performance on immediate word recall than the emigrants who moved from Guangdong to Hong Kong before the famine.

The results of our analyses suggest that the people who experienced the famine during childhood were adversely influenced by the famine in terms of adult cognitive performance, whereas the survivors who were exposed to famine in utero might be positively selected (in utero exposure was so critical that only the positively selected could survive). In other words, while the famine had scarring effects on the survivors, it might, at the same time, have removed those with poor conditions leading to a positive selection of survivors. If the analysed sample in our study was positively selected, the negative effect of famine on cognition might be underestimated.

These findings of our study imply that, as shown in previous studies, brain function is shaped in early life [9,10,20]. The deprived nutrients in childhood negatively affect brain growth and development trajectories, which may have a long-term impact on individuals’ cognitive performance. 

There are several contributions made by this paper. First, it calls for a more nuanced analysis of the differentiated effects of malnutrition at different life stages on adult cognitive performance. This study extended the scope of Becker’s foetal origins hypothesis by showing that malnutrition experienced during childhood and early adolescence also had adverse consequences for cognitive performance. Second, while previous studies found that exposure to the Chinese Famine during the prenatal period created a greater risk of cognitive impairment [11,25,33], our results suggest that severe nutritional deprivation at age 1–18 has a long-term impact on cognition, which was understudied in the previous literature. Our findings about the effects of malnutrition experienced during different life stages on adult cognitive performance offer a significant supplement to the previous literature. Against the background of post-pandemic uncertainties across the globe, the findings of this study suggest a call for effective policies to address the concerns of children and adolescents regarding malnutrition.

Due to data limitations, this study used vocabulary tests and word recall tests to measure cognitive performance. As brain structures affect different indicators of cognitive abilities, the use of multi-dimensional measures would provide a more comprehensive assessment of malnutrition experienced at different life stages and its corresponding impacts on later cognitive performance. We expect future studies with appropriate data to explore the effects of early life adverse experiences on different measures of adult cognitive abilities.

## 5. Conclusions

Our study chose the Chinese Famine as an empirical setting to examine the long-term effects of early life malnutrition on adult cognitive performance. Based on an analysis of the 2010 China Family Panel Studies data, we found that people who experienced the famine in early childhood (aged 1–3) had lower scores on the vocabulary test, and those who were exposed to the famine in utero did not differ from those born after the famine. Based on comparable data from the 2016 CFPS and 2017 HKPSSD, our migrant–stayer comparison analysis further showed that the Guangdong-stayers born between 1941 and 1958 (aged 1–18 when the famine started) had worse cognitive performance in immediate word recall than if they had avoided the famine. Furthermore, these two analyses suggest that the higher the individual’s educational level, the higher their cognitive performance. The findings of this study confirm that exposure to malnutrition during childhood has a long-term impact on individuals’ cognitive performance.

## Figures and Tables

**Table 1:** Long-term famine effect on people’s vocabulary test (the cohort comparisons approach: DID analysis).

	Vocabulary Test
	(1)
Cohort (Ref: post-famine cohort)	
Famine cohort	−0.579
	(0.480)
Pre-famine cohort	0.307
	(0.702)
Famine severity	−0.012
	(0.011)
Cohort × Famine severity	
Famine cohort × famine severity	−0.020
	(0.019)
Pre-famine cohort × famine severity	−0.054 **
	(0.017)
Age	−0.387 ***
	(0.077)
Male	3.095 ***
	(0.197)
Currently married	1.473 ***
	(0.445)
Educational level (Ref: Primary and below)	
Junior high	9.128 ***
	(0.223)
Senior high and above	15.135 ***
	(0.288)
Parents’ educational level (Ref: Primary and below)	
Junior high	0.130
	(0.369)
Senior high and above	2.046 ***
	(0.472)
Parents with party membership	0.562 *
	(0.261)
Urban residence	1.132 ***
	(0.206)
Constant	25.389 ***
	(3.521)
Observations	5572
R-squared	0.482

Notes: Standard errors in parentheses, *** *p* < 0.001, ** *p* < 0.01, * *p* < 0.05.

**Table 2:** OLS regressions of immediate word recall for different birth cohorts (the migrant–stayer comparisons approach).

	1923–1940	1941–1958	1963–1968
	(1)	(2)	(3)
Migrant status (Ref: GD-HK emigrants)			
GD stayers	−0.638 +	−0.646 *	0.340
	(0.337)	(0.321)	(0.296)
Age	−0.013	−0.042 *	−0.127
	(0.036)	(0.017)	(0.078)
Male	0.218	0.126	−0.182
	(0.307)	(0.176)	(0.200)
Currently married	0.046	0.224	0.767 *
	(0.322)	(0.237)	(0.363)
Educational level (Ref: Primary and below)			
Junior high	−0.127	0.199	0.450 *
	(0.940)	(0.241)	(0.209)
Senior high and above	0.562	0.898 ***	0.761 *
	(0.655)	(0.261)	(0.339)
Urban residence	−0.682 +	0.047	0.632 **
	(0.381)	(0.168)	(0.213)
Constant	4.290	6.466 ***	9.306 *
	(3.083)	(1.256)	(4.092)
Observations	130	460	225
R-squared	0.055	0.061	0.106

Notes: Standard errors in parentheses, *** *p* < 0.001, ** *p* < 0.01, * *p* < 0.05, + *p* < 0.1.

**Table 3:** OLS regressions of delayed word recall for different birth cohorts (the migrant-stayer comparisons approach).

	1923–1940	1941–1958	1963–1968
	(1)	(2)	(3)
Migrant status (Ref: GD-HK emigrants)			
GD stayers	−0.461	−0.564	−0.397
	(0.355)	(0.358)	(0.395)
Age	0.005	−0.037 *	−0.038
	(0.038)	(0.018)	(0.104)
Male	0.389	0.244	−0.273
	(0.325)	(0.195)	(0.269)
Currently married	−0.190	0.053	1.737 ***
	(0.341)	(0.260)	(0.500)
Educational level (Ref: Primary and below)			
Junior high	−0.590	0.219	0.202
	(1.003)	(0.274)	(0.283)
Senior high and above	0.043	0.967 **	0.583
	(0.699)	(0.293)	(0.453)
Urban residence	−0.234	0.242	0.710 *
	(0.392)	(0.188)	(0.288)
Constant	1.437	4.825 ***	3.096
	(3.247)	(1.397)	(5.473)
Observations	135	457	219
R-squared	0.031	0.056	0.114

Notes: Standard errors in parentheses, *** *p* < 0.001, ** *p* < 0.01, * *p* < 0.05.

**Table 4:** Difference-in-differences estimates of famine effects on word recall (the migrant–stayer comparisons approach).

	Immediate Word Recall		Delayed Word Recall	
	1923–1940 vs. 1963–1968	1941–1958 vs. 1963–1968	1923–1940 vs. 1963–1968	1941–1958 vs. 1963–1968
Migrant status (Ref: GD-HK emigrants)				
GD stayers	0.300	0.252	−0.307	−0.365
	(0.284)	(0.295)	(0.350)	(0.347)
Birth cohort (Ref: 1963–1968)				
1923–1940	−0.212		−1.511	
	(1.031)		(1.263)	
1941–1958		0.756 +		0.209
		(0.454)		(0.532)
Migrant status × birth cohort				
GD stayers × 1923–1940	−0.597		0.123	
	(0.375)		(0.460)	
GD stayers × 1941–1958		−0.806 *		−0.101
		(0.397)		(0.467)
Age	−0.030	−0.047 **	0.002	−0.038 *
	(0.031)	(0.015)	(0.038)	(0.018)
Male	−0.006	0.047	−0.023	0.090
	(0.165)	(0.132)	(0.204)	(0.156)
Educational level (Ref: Primary and below)				
Junior high	0.420 *	0.342 *	0.219	0.284
	(0.209)	(0.165)	(0.263)	(0.197)
Senior high and above	0.706 *	0.877 ***	0.511	0.906 ***
	(0.305)	(0.207)	(0.376)	(0.245)
Urban residence	0.287	0.214	0.453 +	0.374 *
	(0.190)	(0.133)	(0.234)	(0.158)
Constant	5.133 **	6.046 ***	2.579	4.613 ***
	(1.644)	(0.853)	(2.012)	(1.003)
Observations	355	685	354	676
R-squared	0.252	0.087	0.136	0.064

Notes: Standard errors in parentheses, *** *p* < 0.001, ** *p* < 0.01, * *p* < 0.05, + *p* < 0.1.

## Data Availability

All the data that support the findings of this study are available from the authors upon reasonable request.

## Appendix A

**Table A1:** Summary statistics for selected variables of CFPS 2010 (the cohort comparisons approach).

	Pre-Famine	Famine	Post-Famine	ANOVA/Chi-Squared Test
Vocabulary test	12.1 (10.12)	15.1 (9.64)	16.3 (9.51)	0.015
Age	53.5 (1.19)	49.3 (1.21)	45.5 (1.32)	<0.001
Male (%)	49.8	48.1	47.8	0.495
Currently married (%)	93.7	95.4	96.0	0.004
Educational level (%)				
Primary and below	59.4	45.1	51.2	
Junior high	25.1	31.6	36.8	<0.001
Senior high and above	15.5	23.4	12.1	
Parental educational level (%)				
Primary and below	91.8	89.7	84.4	
Junior high	5.4	6.3	9.6	<0.001
Senior high and above	2.8	4.0	5.9	
Parents with party membership (%)	14.9	17.3	17.4	0.073
Urban residence	35.0	32.6	33.9	0.361
*N*	1772	1403	2397	5572

Notes: Mean and standard deviation are reported. Pre-famine stands for those born during 1955–1958; famine stands for those born during 1959–1962; post-famine stands for those born during 1963–1966.

**Table A2:** Summary statistics for selected variables of the CFPS and HKPSSD combined sample (the migrant–stayer comparisons approach).

	1923–1940	1941–1958	1963–1968
	Mean (%)	Mean (%)	Mean (%)
Migrant status (%)			
Guangdong-Hong Kong (GD-HK) emigrants	48.5	8.3	18.2
Guangdong (GD) stayers	51.5	91.7	81.8
Dependent variables			
Immediate word recall	2.5 (1.52)	3.5 (1.76)	4.2 (1.45)
Delayed word recall ^a^	1.5 (1.61)	2.3 (1.94)	2.8 (1.94)
Control variables			
Age	82.6 (4.15)	65.4 (5.16)	51.7 (1.28)
Male (%)	53.1	53.7	45.3
Educational level (%)			
Primary and below	93.1	73.9	56.4
Junior high	2.3	14.4	32.9
Senior high and above	4.6	11.7	10.7
Currently married (%)	60	85.9	92.4
Urban residence	77.7	49.1	49.8
*N*	130	460	225

Notes: Mean and standard deviation are reported. ^a^ In the delayed word recall analysis, the sample sizes were 135, 457, and 219, respectively, for the 1923–1940, 1941–1958, and 1963–1968 cohorts.

**Table A3:** Long-term famine effect on people’s vocabulary test (the cohort comparisons approach).

	Vocabulary Test
	(1)
Cohort (Ref: 1963–1968)	
1923–1940	−5.328 ***
	(0.653)
1941–1958	−2.525 ***
	(0.357)
Famine severity	−0.014
	(0.009)
Cohort × Famine severity	
1923–1940 × Famine severity	−0.022
	(0.018)
1941–1958 × Famine severity	−0.066 ***
	(0.012)
Age	0.030 +
	(0.018)
Male	4.372 ***
	(0.146)
Currently married	1.567 ***
	(0.240)
Educational level (Ref: Primary and below)	
Junior high	9.411 ***
	(0.186)
Senior high and above	15.004 ***
	(0.261)
Parents’ educational level (Ref: Primary and below)	
Junior high	0.493
	(0.310)
Senior high and above	1.804 ***
	(0.387)
Parents with party membership	0.552 *
	(0.224)
Urban residence	2.010 ***
	(0.151)
Constant	5.722 ***
	(0.892)
Observations	11,543
R-squared	0.462

Notes: Standard errors in parentheses, **** p <* 0.001, ** p <* 0.05, *+ p <* 0.1.
